# Happy Family Kitchen II: A Cluster Randomized Controlled Trial of a Community-Based Family Intervention for Enhancing Family Communication and Well-being in Hong Kong

**DOI:** 10.3389/fpsyg.2016.00638

**Published:** 2016-05-03

**Authors:** Henry C. Y. Ho, Moses Mui, Alice Wan, Yin-Lam Ng, Sunita M. Stewart, Carol Yew, Tai Hing Lam, Sophia S. Chan

**Affiliations:** ^1^School of Public Health, The University of Hong KongHong Kong, China; ^2^Service Development, The Hong Kong Council of Social ServiceHong Kong, China; ^3^Department of Psychiatry, The University of Texas Southwestern Medical Center at DallasDallas, TX, USA; ^4^United Centre of Emotional Health and Positive Living, United Christian Nethersole Community Health ServiceHong Kong, China; ^5^School of Nursing, The University of Hong KongHong Kong, China

**Keywords:** randomized controlled trial, community-based intervention, family communication, family well-being, positive psychology

## Abstract

Long working hours and stressful urban lifestyles pose major challenges to family communication and well-being in Hong Kong. A community-based family intervention derived from a positive psychology framework, by using cooking and dining as a platform, was developed for improving family communication and well-being. Social workers and teachers from 31 social service units and schools in collaboration with an academic partner organized and conducted the intervention programs for 2,070 individuals from 973 families in a deprived district in Hong Kong. The participants were randomly assigned into the intervention or control group in a cluster randomized controlled trial (cRCT). The core intervention covered one of five positive psychology themes: joy, gratitude, flow, savoring, and listening. Assessments at pre-intervention, immediate post-intervention, and 4 and 12 weeks post-intervention showed improved family communication and well-being with sustainable effects up to 12 weeks. Positive changes in family happiness and family health were greater in the intervention group than in the control group. The savoring intervention had the most improved outcomes among the five themes. We concluded that this large-scale brief cRCT developed and conducted in real-world settings provided evidence for the feasibility and effectiveness of a community-based family intervention. This study was registered under ClinicalTrials.gov (NCT01796275).

## Introduction

Long working hours and stressful urban lifestyles pose major challenges to families in establishing positive communication and maintaining family gatherings that are fundamental for the bonding of family members (Lizano et al., 2014; Winefield et al., 2014). With regard to these concerns, the Happy Family Kitchen (HFK I) project was conducted before the present Happy Family Kitchen II (HFK II) project, to develop, implement, and evaluate a community-based family intervention program for improving family communication and family well-being in Hong Kong. HFK I was one of the major intervention projects under The FAMILY Project, which aimed to promote family health, happiness, and harmony (Stewart et al., 2012). The intervention was developed with reference to positive psychology as a guiding framework and cooking and dining with family members as a platform. We used a one-group pretest and repeated post-test design over a period of 12 weeks, with programs covering one of five positive psychology themes: gratitude, flow, happiness, health, and savoring. The results showed that the overall intervention program was effective in improving family communication and well-being. Furthermore, the gratitude and happiness interventions appeared to be the most effective on the outcome measures, whereas the health intervention was the least effective. To extend our previous work, we made three major improvements in the HFK II project: (a) We enhanced the practice model by replacing the health theme with the listening theme; (b) we enhanced the scientific rigor by adopting a cluster randomized controlled trial (cRCT); and (c) we increased the public health impact by recruiting a larger sample from different districts in Hong Kong.

An emphasis on positive communication, positive emotions, and the appreciation of family strengths has been recommended for effective family interventions (Sexton and Schuster, 2008; Kauffman and Silberman, 2009). Another previous study in The FAMILY Project reported that gratitude, hope, and open-mindedness were effective themes for promoting theme-related attitudes, intentions, behaviors, and family well-being in a community-based family intervention (Zhou et al., 2016). In the design of the current HFK II project, we were guided by several factors to expand on prior research. For feasibility, practicality, and cultural relevance, we continued to use cooking and dining as a platform to promote communication and positive interactions. These family based activities would not add time demands to people who face conflicts between work and family life. Furthermore, in Chinese culture, cooking and dining with family members is emphasized and provides a channel to promote family bonding and the transference of knowledge and skills between generations (Wong, 2010; Lai-Yeung, 2015). Food sharing symbolizes family cohesiveness and helps with the reaffirmation of family relationships. For acceptability and sustainability, most intervention programs require intensive and extensive involvement from both the service providers and service recipients (Seligman et al., 2006; Ho et al., 2014). This results in a high demand for manpower and other resources, making such intervention programs difficult to sustain in large community contexts. Therefore, the current intervention was brief, consisting of a core session and a booster session, which minimized program implementation costs as well as the time burden on participants. Finally, we considered that an intervention program that applied the hands-on experiential learning of principles relevant to our outcomes would engage participants more compared to didactic education.

Our intervention emphasized positive communication within the contexts of cooking and dining by using a positive psychology approach (Sheridan et al., 2004; Sexton and Schuster, 2008; Kauffman and Silberman, 2009). Positive psychology—the study of character strengths and virtues—is instrumental in conceptualizing services that promote family communication and well-being through positive subjective experiences (e.g., satisfaction and happiness) and character strengths (e.g., gratitude and love) within the context of positive institutions (e.g., family; Seligman and Csikszentmihalyi, 2000; Seligman et al., 2006; Nicoll, 2011; Morganson et al., 2014). Although positive subjective experiences and character strengths have often been viewed at an individual level, they are also critical for the adaptive functioning of family relationships by buffering against family problems and supporting families through difficulties (Shapiro, 2004). Rather than to rectify family problems, our intervention aimed to promote family communication and well-being by using existing family strengths, emphasizing family identified needs, and acquiring new skills and competencies (Sheridan et al., 2004).

Five positive psychology themes were adopted: joy, gratitude, flow, savoring, and listening. Brief descriptions of the themes are summarized in **Table 1**. To cultivate positive emotions among family members, the “joy” theme was used to facilitate communication by emphasizing the short-term pleasures and long-term gratifications that family interactions can bring (Seligman, 2002). The expression of thankfulness and appreciation toward family members were nurtured in the “gratitude” theme (Peterson and Seligman, 2004). The “flow” theme, which fully engages with family members in an activity, was incorporated to help family members to discover each other’s strengths, increase interaction, and encourage mutual cooperation so that the activity is fully engrossing and enjoyable (Csikszentmihalyi, 1997). The “savoring” theme was used to nurture the ability to fully enjoy the present moment and everyday life experiences, particularly when dining with family members (Seligman et al., 2006). Finally, the “listening” theme emphasized the importance of social and emotional intelligence, which involves paying attention to family members’ views and concerns, being attentive to their emotions, and responding appropriately to their needs (Seligman et al., 2004).

**Table 1:** Description of the Happy Family Kitchen II Program.

Theme	Purpose	Suggested activities and homework assignments
Joy	The purpose of the joy intervention was to cultivate happiness through positive communication with family members.	Activity 1: participants were encouraged to share and reminisce about their happy experiences with family members and create more happy experiences by enjoying a meal together.Activity 2: each family member wrote down the things that are most commonly said and heard at home and discussed how the negative messages can be rephrased.Homework 1: share a happy experience during family dinner every day and keep a daily diary of the sharing.Homework 2: avoid the negative communication style at home.
Gratitude	The gratitude intervention was intended to nurture a habit of expressing gratitude and appreciation toward family members, especially for the preparation of family meals.	Activity: participants discussed about their family members’ contribution to family meals and other chores and expressed appreciation through words or action.Homework: write down gratifying and praiseful messages toward family members and store them in a collection box for sharing.
Flow	In the flow intervention, the goal was to identify each other’s strengths, increase interaction, and encourage mutual cooperation.	Activity: participants learned to prepare a family meal together, and through this process, learned to cooperate and recognize each other’s strengths.Homework: take photos of family gatherings to keep a record of events that involved the contribution of each family member.
Savoring	In the savoring intervention, the goal was to nurture a habit of savoring food prepared by family members and treasuring the time during family meals.	Activity 1: blindfolded participants were spoon-fed by their family member and were asked to guess what was fed to them to emphasize the importance of savoring and communication.Activity 2: participants were asked to guess the ingredients of several specified dishes and were explained the importance of savoring and respect during family meals.Homework 1: keep a record of family meals that start and end together.Homework 2: take time to learn family recipes from a family member who cooks regularly so that his or her efforts are understood.
Listening	The listening intervention focused on active listening skills so that family members’ feelings, emotions, and concerns can be understood.	Activity 1: participants played a game that involved matching emotion adjectives so that they can better express and respond to emotions during family meals.Activity 2: participants took turns to read a hypothetical passage that described their emotions of the day, and their family members were instructed to respond appropriately by considering their thoughts, feelings, and concerns.Homework 1: keep a diary that records the emotions of family members every day.Homework 2: practice active listening every day.

We hypothesized that (H1) participants in the intervention arm would show improved family communication and family well-being, including health, happiness and harmony; (H2) the pre–post effect size of the intervention group would be larger than that of the control group; (H3) each of the five thematic programs would show improvements in the four outcome measures; and (H4) the booster session would be effective on the four outcome measures at 12 weeks after the baseline assessment. To our knowledge, no similar studies have been conducted using a large-scale cRCT design to examine the effectiveness of a community program on family communication and well-being.

## Materials and Methods

### Participants

Participants were recruited from local social service organizations, the Social Welfare Department of the Hong Kong Special Administrative Region, as well as primary and secondary schools in the Tsuen Wan and Kwai Tsing districts in Hong Kong. These two districts were selected because they are more socially and economically deprived in Hong Kong and consist of more underprivileged families (Census and Statistics Department, 2013). For greater public health impact, the community program was targeted at the general population (Spoth et al., 2002). The selection criteria of this study were: (a) residents, service users, or students in the Tsuen Wan or Kwai Tsing district; (b) willing to participate with one or more family members; (c) at least one family member was aged 18 years or older and the accompanying family member(s) was aged 6 years or older; and (d) were able to communicate in Chinese. On the basis of prior experience in the HFK I project, a sample size of at least 1,920 was required for this cRCT to detect small effect sizes (ES = 0.20), with a statistical power of 0.80, an alpha of 0.05, and an attrition rate of 50% (Hemming et al., 2011). A total of 2,513 individuals were invited, and 2,070 eligible participants from 973 families participated in the study.

Different recruitment methods were used by the participating service organizations and schools to approach and attract potential participants, including (a) phone invitations; (b) promotional materials such as posters, banners, leaflets, and publications; (c) promotion through e-mails and websites; (d) face-to-face invitations; (e) social workers’ and teachers’ referrals; (f) outreach recruitment on the streets; and (g) home visits. Written consent was obtained from each participant prior to the programs. For children enrolled in the study, written consent was obtained from the next of kin, caretakers, or guardians on their behalf. Participation was completely voluntary and participants had the right to withdraw at any time without consequences. As an incentive for completing all four questionnaires, two HK$50 (approximately US$13) supermarket gift vouchers were given to each family at the end of the study. Ethics approval was granted by the Institutional Review Board of the University of Hong Kong/Hospital Authority Hong Kong West Cluster (UW 12-502). This study was registered under ClinicalTrials.gov (NCT01796275).

### Procedures

Social service workers and teachers from the participating service organizations and schools were trained to deliver the programs. The train-the-trainer workshop was delivered by professional academics and psychologists to comprehensively cover the contents of the five themes of positive psychology, the program design, and the program evaluation. A training kit was distributed to each trainee as a practical guide for planning the community programs. The evaluation of the workshop is reported elsewhere as it is beyond the scope of this paper.

**FIGURE 1:**
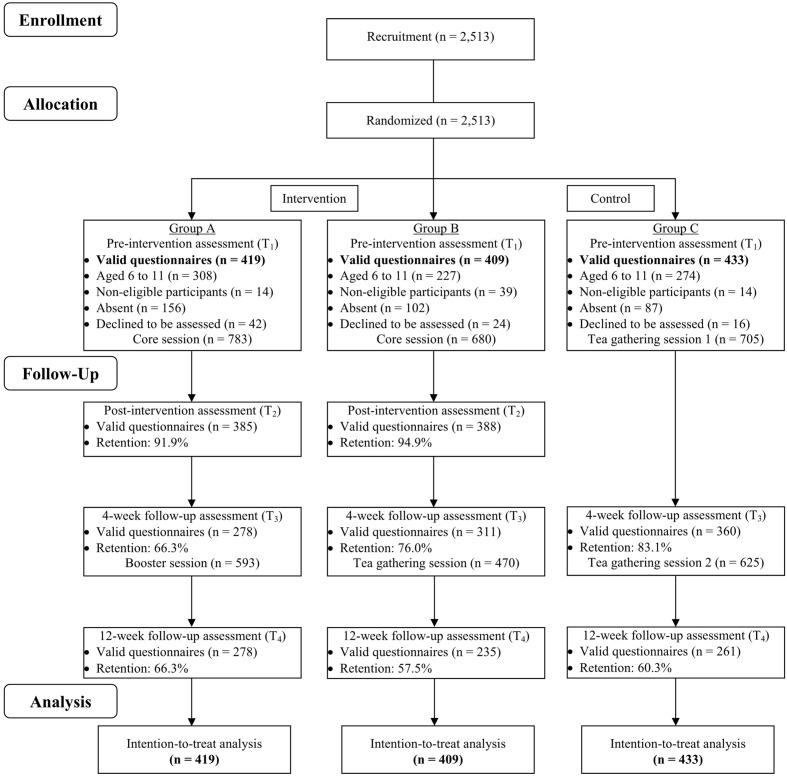
**The Consolidated Standards of Reporting Trials (CONSORT) flow diagram of participants in Happy Family Kitchen II through each stage of the study.** The core program contents of Groups A and B were identical.

A total of 31 social service units and schools organized and conducted the programs. These social service units and schools were treated as clusters in a cRCT, such that the clusters of participants were randomly allocated into three groups by using computer-generated random numbers. Randomization and allocation were performed by an independent statistician who had no contact with the organizations, schools, or participants. Group A (intervention arm 1) received a core session of at least 2 h, followed by a booster session of at least 1 h 4 weeks later; Group B (intervention arm 2) also received a core session of at least 2 h followed by a tea gathering session 4 weeks later (no booster session); Group C, the control arm (waitlist control), had a tea gathering session at the beginning and 4 weeks later. Specifically, the core session involved group activities and homework assignments focusing on a positive psychology theme; the booster session focused on consolidating the knowledge and skills obtained from the core session; the tea gathering session covered topics unrelated to the intervention, such as arts and crafts workshops. For outcome assessment, participants completed a self-administered questionnaire in the service settings at four time points: pre-intervention (baseline assessment, T_1_), immediate post-intervention (immediately after the core session for Groups A and B only, T_2_), 4 weeks after the baseline assessment (before the booster session in Group A, before the tea gathering in Group B, and before the second tea gathering in Group C, T_3_), and 12 weeks after the baseline assessment (T_4_). The Consolidated Standards of Reporting Trials (CONSORT) flow diagram is presented in **Figure 1**.

Since a large number of the participants (*n* = 809) were aged 6–11 years, cognitive interviews were conducted to assess their understanding of the questionnaire items prior to the study. Following the cognitive psychological theory, these qualitative interviews aimed to reveal hidden aspects of the survey response process (Tourangeau, 1984). Interviews with 35 individuals from this age group showed that their ability to comprehend the questions varied vastly and the wording of the questions could be too vague for the younger participants. In particular, those who were aged 6–8 years had the most difficulty in completing the questionnaires by themselves. Therefore, staff and volunteers from the participating service organizations and schools were suggested to conduct individual face-to-face interviews to assist the younger participants in questionnaire completion.

### Intervention Program

Social service workers and teachers from the participating service organizations and schools were trained to design and implement the community programs by focusing on one of the five positive psychology themes they had chosen. The outcomes to be achieved were the same for all programs regardless of the themes. Positive family communication was emphasized in all themes by using family cooking and dining as a platform. The collaborative involvement of practitioners and academics in program design and implementation promoted the sharing of expertise between professionals from different disciplines, facilitated participant recruitment, intervention implementation, and data collection at a community level, and enhanced the future dissemination of the intervention program if it is proven effective (Stewart et al., 2012). Furthermore, by allowing the interventionists to flexibly select a positive psychology theme and design a custom-tailored community program in accordance with the common guiding principles and objectives, the unique needs of families were accommodated. In our previous study (Zhou et al., 2016), we found that this approach was effective for people with diverse demographic characteristics.

Each eligible participant in the intervention group received one positive psychology theme of the community program. The use of themes in Groups A and B was as follows: 6 used joy [number of eligible participants (*n* = 333), 3 used gratitude (*n* = 279), 4 used flow (*n* = 249), 4 used savoring (*n* = 280), and 4 used listening (*n* = 222)]. To ensure adherence to the guiding principles so that the consistency and quality of the community programs were maximized, participating service organizations and schools submitted program proposals to the project steering committee, received comments and made improvements accordingly, and then were awarded funding to implement the proposals. The project steering committee consisted of academic researchers and managerial staff from the participating service organizations and schools. Standardized process evaluation was conducted by a research assistant through the onsite observation of each session (core session, booster session, and tea gathering) to assess fidelity (measured by adherence to program guidelines), the dose delivered (measured by the duration of program delivery), and the dose received (measured by the participants’ interest, involvement, and satisfaction in the program). Overall, 81.8 and 72.7% of the community programs were implemented in accordance with the specified fidelity and dosage, respectively. On a five-point scale, the participants’ interest (*M* = 4.10, *SD* = 0.59), involvement (*M* = 4.16, *SD* = 0.68), and satisfaction (*M* = 4.14, *SD* = 0.58) in the programs were high.

### Primary Outcome Measures

#### Family Communication Scale

This 10-items scale was used to assess the most important aspects of communication in a family system at T_1_, T_3_, and T_4_ (Olson and Barnes, 2004). Responses were made on a five-point scale, ranging from 1 (*strongly agree*) to 5 (*strongly disagree*), with a higher total score indicating more positive family communication after reverse coding. An example of the scale is “Family members are satisfied with how they communicate with each other.” The Cronbach’s alpha ranged from 0.91 to 0.93 across three time points.

#### Family Health, Happiness, and Harmony

To assess family well-being from T_1_ to T_4_, three single item indicators of family health, happiness, and harmony were used (Wang et al., 2014). Responses were made on an 11-point scale, ranging from 0 (*not at all*) to 10 (*very much*), with higher scores indicating higher levels of family well-being. The family health question was “Do you think your family is healthy?” The family happiness question was “Do you think your family is happy?” The family harmony question was “Do you think your family is harmonious?”

### Data Analysis

WinBUGS (Lunn et al., 2000) was used for data analysis because it allows flexible statistical models and prior distributions to be fitted, and provides transparent diagnostics for checking MCMC convergence and model fit. To examine the effectiveness of the community programs, random effects linear models were fitted to the outcomes of interest (i.e., family communication and well-being) for the three groups (i.e., Groups A, B, and C), four time points (i.e., T_1_, T_2_, T_3_, and T_4_), and five themes (i.e., joy, gratitude, flow, savoring, and listening) while controlling for possible confounding effects of age, gender, and education level. Furthermore, individual correlations across time, correlations among individuals within a family unit, and the cluster effects of individuals within the same program session were treated as random effects with different variances. These analytical procedures were suitable for assessing whether there were intervention effects over time (i.e., T_2_–T_1_, T_3_–T_1_, and T_4_–T_1_), whether there were differences in the outcome changes between the intervention and control groups (i.e., A & B vs. C), whether there were any individual impacts of each of the five thematic interventions, and whether the booster session had any effect on the outcome measures (i.e., A vs. B in T_4_–T_1_). Because of the questionable validity of the responses from young participants, the main analyses reported here excluded data from those who were aged 6–11 years (remaining sample = 1,261). Sensitivity analysis with young participants included produced similar results for most hypotheses (tables not shown).

Analysis was conducted with intention-to-treat (Fisher et al., 1990), adopting a full maximum likelihood inference with the assumption that data were missing at random, such that individuals with missing data were assumed to behave similarly to individuals with complete data and similar demographic characteristics (Rubin, 1976). This assumption was more realistic than assuming that data were missing completely at random. It was also preferred to the baseline or last observation carried forward analyses because uncertainties caused by missing observations were taken into account to generate more reliable intervention estimates with statistically robust standard errors (Schafer and Graham, 2002). Because analysis from the baseline and last observation carried forward methods produced similar results in this study, only the results produced from full maximum likelihood inference are reported here.

Cohen’s *d* was computed with a positive effect size (ES) indicating an increase in the mean score of the outcome, and a negative ES indicating a decrease. An ES of 0.2 was considered as a small effect, 0.5 as a medium effect, and 0.8 or above as a large effect (Cohen, 1977).

## Results

### Sample Characteristics

Overall, the majority of the eligible participants were women (64.6%). The age distribution was 6–8 years (24.9%), 9–13 years (17.3%), 14–17 years (1.9%), 18–34 years (13.2%), 35–44 years (25.5%), 45–54 years (9.7%), 55–64 years (3.4%), and 65 years or older (2.0%). Most of them had primary (46.0%) or secondary (42.6%) education level. Group C participants were more likely to be 18 years or older [56.2%, χ^2^(2) = 75.2, *p* < 0.001] and least likely to have received tertiary education or above [5.0%, χ^2^(2) = 48.7, *p* < 0.001, **Table 2**]. Furthermore, they scored higher on family health at baseline [*M* = 7.96, *SD* = 1.95; *F*(2,2067) = 4.00, *p* = 0.02] compared to Group A (*M* = 7.73, *SD* = 2.15) and Group B (*M* = 7.64, *SD* = 2.16).

**Table 2:** Demographic characteristics and outcome measures at baseline.

		Group A (*n* = 727)	Group B (*n* = 636)	Group C (*n* = 707)	*p*-value^e^
Age^a,b^					<0.001***
	6–8	192 (26.7)	136 (22.0)	187 (27.1)	
	9–13	140 (19.4)	113 (18.3)	106 (15.4)	
	14–17	6 (0.8)	26 (4.2)	8 (1.2)	
	18–34	60 (8.3)	95 (15.3)	118 (17.1)	
	35–44	195 (27.1)	147 (23.7)	186 (27.0)	
	45–54	77 (10.7)	72 (11.6)	52 (7.5)	
	55–64	24 (3.3)	24 (3.9)	23 (3.3)	
	65 or older	26 (3.6)	6 (1.0)	9 (1.3)	
Gender^a,b^					0.34
	Male	255 (35.1)	239 (37.6)	239 (33.8)	
	Female	472 (64.9)	397 (62.4)	468 (66.2)	
Education level^a,b^					<0.001***
	None	15 (2.1)	8 (1.3)	8 (1.2)	
	Primary	373 (52.1)	269 (43.7)	311 (45.5)	
	Secondary	280 (39.1)	271 (44.1)	331 (48.4)	
	Tertiary or above	48 (6.7)	67 (10.9)	34 (5.0)	

Family communication^c,d^		67.07 (17.36)	66.79 (17.22)	67.53 (17.77)	0.74
(10 items, 0–100)						
Family health^c,d^		7.73 (2.15)	7.64 (2.16)	7.96 (1.95)	0.02*
(one item, 0–10)						
Family happiness^c,d^		7.52 (2.19)	7.53 (2.18)	7.60 (2.21)	0.74
(one item, 0–10)						
Family harmony^c,d^		7.65 (2.15)	7.51 (2.25)	7.60 (2.19)	0.50
(one item, 0–10)						

*^a^n (%).*

*^b^p-values generated from the Pearson chi-square test.*

*^c^M (SD).*

*^d^p-values generated from ANOVA.*

^e*^p < 0.05; **p < 0.01; ***p < 0.001.

### Outcomes by Time Point

For H1, Groups A and B were pooled together as the intervention group because the core program objectives were similar. Significant improvements for all outcome measures were observed (**Table 3**). In particular, family communication increased significantly at T_3_ (ES = 0.16, *p* < 0.05) and T_4_ (ES = 0.18, *p* < 0.05) compared to baseline. We also found that family health increased significantly at T_2_ (ES = 0.47, *p* < 0.001) and T_4_ (ES = 0.17, *p* < 0.05), family happiness at T_2_ (ES = 0.67, *p* < 0.001), T_3_ (ES = 0.17, *p* < 0.05), and T_4_ (ES = 0.36, *p* < 0.001), as well as family harmony at T_2_ (ES = 0.48, *p* < 0.001) and T_4_ (ES = 0.17, *p* < 0.05). By contrast, there were no significant improvements in the control group on all outcome measures at T_3_ and T_4_.

**Table 3:** Changes in Groups A and B on family communication and well-being.

	Difference in time point	Difference in *M* (*SD*)	ES^b,c^
Family communication^a^(10 items, 0–100)	T_3_–T_1_	1.15 (0.50)	0.16*
	T_4_–T_1_	1.28 (0.51)	0.18*
Family health(one item, 0–10)	T_2_–T_1_	0.40 (0.06)	0.47***
	T_3_–T_1_	0.08 (0.06)	0.09
	T_4_–T_1_	0.15 (0.06)	0.17*
Family happiness(one item, 0–10)	T_2_–T_1_	0.56 (0.06)	0.67***
	T_3_–T_1_	0.15 (0.06)	0.17*
	T_4_–T_1_	0.32 (0.06)	0.36***
Family harmony(one item, 0–10)	T_2_–T_1_	0.38 (0.05)	0.48***
	T_3_–T_1_	0.01 (0.06)	0.01
	T_4_–T_1_	0.14 (0.06)	0.17*

*Group A (*n* = 419); Group B (*n* = 409).*

*Excluded young participants aged < 12 years.*

^a^Family communication was not assessed at T_*2*_.

*p < 0.05; **p < 0.01; ***p < 0.001.

*^c^Effect size (ES) = Cohen’s d (small = 0.20; medium = 0.50; large = 0.80).*

### Effectiveness by Group

**FIGURE 2:**
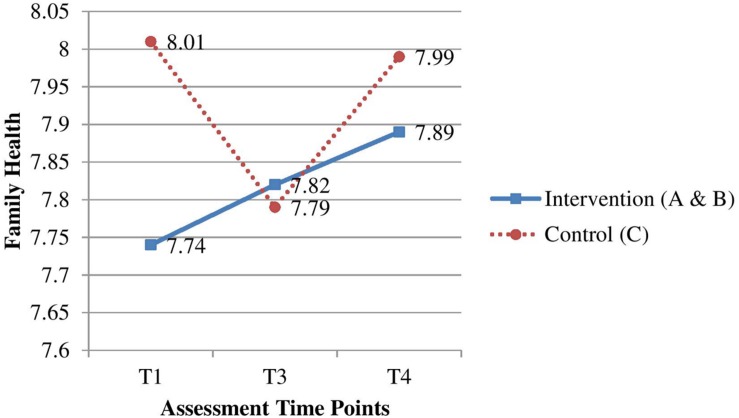
**Effectiveness of the intervention group on family health compared with the control group**.

**FIGURE 3:**
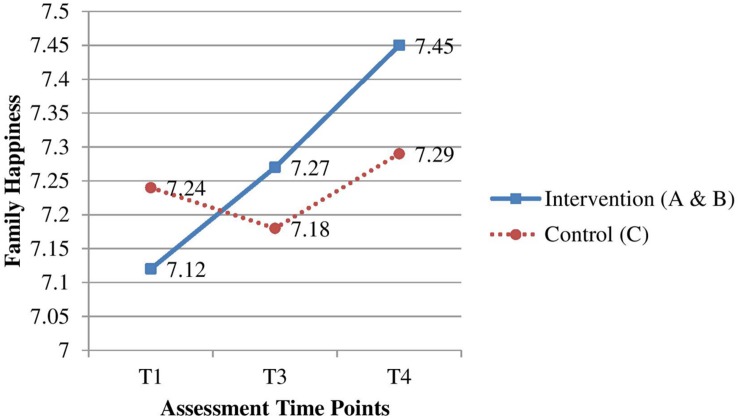
**Effectiveness of the intervention group on family happiness compared with the control group**.

For H2, the joint changes in Groups A and B at T_3_ and T_4_ after baseline were compared with changes in Group C (**Table 4**). The improvements for family health at T_3_ (ES = 0.20, *p* < 0.01, **Figure 2**) and family happiness at T_3_ and T_4_ (ES = 0.14, *p* < 0.05; ES = 0.18, *p* < 0.01, respectively, **Figure 3**) were significantly greater in the intervention group than in the control group. However, improvements on family communication and family harmony in the intervention group did not significantly differ from those in the control group at T_3_ and T_4._

**Table 4:** Changes in Groups A and B compared with Group C on family communication and well-being.

	Time	Groups A and B	Group C	Difference	Change difference	ES^a,b^
	point	*M* (*SD*)	*M* (*SD*)	in time point	*M* (*SD*)	
Family communication(10 items, 0–100)	T_1_	65.55 (1.98)	66.35 (2.08)	-	-	-


	T_3_	66.70 (1.99)	66.61 (2.09)	T_3_–T_1_	0.89 (0.83)	0.07
	T_4_	66.83 (1.99)	67.19 (2.10)	T_4_–T_1_	0.44 (0.87)	0.04
Family health(one item, 0–10)	T_1_	7.74 (0.22)	8.01 (0.22)	-	-	-


	T_3_	7.82 (0.23)	7.79 (0.23)	T_3_–T_1_	0.30 (0.11)	0.20**
	T_4_	7.89 (0.23)	7.99 (0.23)	T_4_–T_1_	0.17 (0.11)	0.11
Family happiness(one item, 0–10)	T_1_	7.12 (0.24)	7.24 (0.25)	-	-	-


	T_3_	7.27 (0.24)	7.18 (0.25)	T_3_–T_1_	0.21 (0.10)	0.14*
	T_4_	7.45 (0.24)	7.29 (0.25)	T_4_–T_1_	0.28 (0.11)	0.18**
Family harmony(one item, 0–10)	T_1_	7.23 (0.23)	7.32 (0.25)	-	-	-


	T_3_	7.24 (0.23)	7.28 (0.25)	T_3_–T_1_	0.04 (0.10)	0.03
	T_4_	7.38 (0.23)	7.29 (0.25)	T_4_–T_1_	0.16 (0.10)	0.11

*Group A (*n* = 419); Group B (*n* = 409); Group C (*n* = 433).*

*Excluded young participants aged < 12 years.*

^a*^p < 0.05; **p < 0.01; ***p < 0.001.

*^b^Effect size (ES) = Cohen’s d (small = 0.20; medium = 0.50; large = 0.80).*

### Effectiveness by Theme

For H3, focusing on the effect size, the savoring intervention improved the most outcomes among the five themes (**Table 5**). Compared to the control group, the savoring intervention was more effective in strengthening family health (T_3_, ES = 0.26, *p* < 0.01) and family happiness (T_3_, ES = 0.22, *p* < 0.05; T_4_, ES = 0.20, *p* < 0.05). The flow intervention was also more effective in strengthening family health (T_3_, ES = 0.23, *p* < 0.05) and family happiness (T_3_, ES = 0.22, *p* < 0.05) compared to the control group. Furthermore, the gratitude intervention showed significantly more improvements in family health (T_3_, ES = 0.29, *p* < 0.01), whereas the joy intervention showed significantly more improvements in family happiness (T_4_, ES = 0.26, *p* < 0.01) compared to the control group. The listening intervention was the least effective among the five themes, with no significant between-group improvements in all outcome measures.

**Table 5:** Changes in Groups A and B compared with Group C on family communication and well-being by theme.

Theme	Outcome measures	Change difference (ES^a,b^)	
		T_3_–T_1_	T_4_–T_1_
Joy	Family communication	0.07	0.06
	Family health	0.03	0.15
	Family happiness	0.08	0.26**
	Family harmony	-0.04	0.17
Gratitude	Family communication	0.11	0.11
	Family health	0.29**	0.04
	Family happiness	0.04	0.07
	Family harmony	0.02	0.13
Flow	Family communication	0.13	-0.06
	Family health	0.23*	0.18
	Family happiness	0.22*	0.19
	Family harmony	0.10	0.16
Savoring	Family communication	0.03	0.04
	Family health	0.26**	0.10
	Family happiness	0.22*	0.20*
	Family harmony	0.07	0.02
Listening	Family communication	-0.01	-0.01
	Family health	0.13	-0.02
	Family happiness	0.08	0.05
	Family harmony	-0.01	0.03

*Group A (*n* = 419); Group B (*n* = 409); Group C (*n* = 433).*

*Excluded young participants aged <12 years.*

^a*^p < 0.05; **p < 0.01; ***p < 0.001.

*^b^Effect size (ES) = Cohen’s d (small = 0.20; medium = 0.50; large = 0.80).*

### Effectiveness of the Booster Session

For H4, changes in Group A at T_4_ after baseline were compared with changes in Group B. Family communication, and family health, happiness, and harmony were not significantly different between Groups A and B at T_4_ (ES = -0.21, ns; ES = 0.04, ns; ES = -0.09, ns; ES = -0.05, ns, respectively).

## Discussion

Across time points, the results showed that the overall intervention program improved family communication (T_3_, T_4_), family health (T_2_, T_4_), family happiness (T_2_, T_3_, T_4_), and family harmony (T_2_, T_4_), thus supporting H1. Most importantly, the intervention was more effective than the control group in improving family health and family happiness, thus partially supporting H2. Furthermore, the savoring intervention appeared to be more effective than the other four themes, with improvements on family health and family happiness, thus partially supporting H3. However, the booster session was not effective on the outcome measures at 12 weeks after the baseline assessment, thus rejecting H4.

This large-scale cRCT extended previous research on positive psychology intervention in three ways. First, our program was a brief intervention that consisted of only one core session. Unlike the intensive multisession protocols that were offered in positive psychology studies (Seligman et al., 2006; Ho et al., 2014), a single session program is less expensive to deliver. Although, a booster session was included in this program, we did not find support for its effectiveness on the outcome measures. Therefore, single session programs could be a low cost and simple approach to enhance family well-being at the community level. Second, our programs were tailored to the needs of families rather than to individuals. Previous positive psychology studies have mainly considered the recipients’ well-being at an individual level, such as life satisfaction, psychological well-being, emotional well-being, resilience, hope, and depression (Sin and Lyubomirsky, 2009; Meyer et al., 2012; Pietrowsky and Mikutta, 2012; Odou and Vella-Brodrick, 2013). By involving family members in a tailor-made intervention with cooking and dining as a platform, improvements in family well-being could be maximized. Third, by applying a positive psychology approach in a community context, this study demonstrated that positive psychology concepts can be operationalized in social service and school settings. We suggest the use of savoring theme because of its effectiveness in community-based family programs.

## Conclusion

Our study had several limitations. First, the cRCT might have led to imbalanced demographic characteristics and baseline measures among the three groups. However, this method was preferable to individual-based randomization because of the reduced risk of experimental contamination and lower administrative costs. Future studies should consider using the minimization method (Pocock and Simon, 1975) in conjunction with a cRCT to achieve balance in stratifying factors. Second, the effect size of the intervention ranged from small to moderate because the intensity of the program was brief. However, in line with the public health approach, brief interventions can enhance the feasibility, retention rate, and population reach at low costs. Third, as with every intervention that involves informed consent, the results might be influenced by self-selection bias. People who join community programs intended to foster family relationships may have more favorable family communication and well-being than those who refrain from joining these programs. There could be a ceiling effect that reduces the effect size of the intervention. Fourth, young participants aged 6–11 years were excluded from the main analyses because of questionable responses. The smaller sample size could reduce the statistical power to detect an effect. Fifth, the response rates at 4 weeks (i.e., Group A) and 12 weeks (i.e., Groups A–C) after the intervention were low. Future studies may consider using more appealing incentives to increase the response rates. Finally, single item measures of family health, happiness, and harmony were used to assess the effectiveness of the programs, which raises concerns about scale reliability. However, there are validity and acceptability advantages to simple and brief questionnaires when the participants come from low socioeconomic backgrounds (Stewart et al., 2012).

Through the joint efforts of researchers and practitioners, the findings from this large-scale cRCT study provide encouraging but non-definitive evidence for the application of a community-based family intervention project that uses a positive psychology framework to promote family communication and well-being. Cooking and dining as a culturally relevant medium provide a viable and attractive approach for improving family functioning in this collectivistic community. Future research should also consider developing and evaluating culturally sensitive family interventions for other collectivist cultures and individualistic communities.

## Author Contributions

HH interpreted the data and wrote the manuscript; MM participated in the design, implementation, and coordination; AW participated in the design, implementation, and coor dination; Y-LN performed the statistical analysis; SS contributed to the conceptualization and writing of the manuscript; CY participated in the design and implementation; THL participated in the design, coordination, and conceptualization and writing of the manuscript; and SC participated in the design, coordination, and conceptualization. All authors read and approved the final manuscript.

## Conflict of Interest Statement

The authors declare that the research was conducted in the absence of any commercial or financial relationships that could be construed as a potential conflict of interest.The reviewer MK and handling Editor declared their shared affiliation, and the handling Editor states that the process nevertheless met the standards of a fair and objective review.
